# FitFoldData: A Web-Based Toolkit for Circular Dichroism and Differential Scanning Calorimetry Data Analysis of Protein Thermal Denaturation

**DOI:** 10.1021/acsomega.5c05567

**Published:** 2025-09-26

**Authors:** Knarik V. Yeritsyan, Artem V. Badasyan

**Affiliations:** Materials Research Laboratory, 119110 University of Nova Gorica, Vipavska 13, SI-5000 Nova Gorica, Slovenia

## Abstract

This work presents FitFoldData, a novel web-based tool for the analysis and interpretation of thermal denaturation data collected from circular dichroism (CD) and differential scanning calorimetry (DSC) experiments. The analytical method employed is based on the recently reported modified Zimm–Bragg model, which includes hydrogen-binding interactions with solvent water. It leverages an intuitive interface, enabling users to efficiently analyze experimental data on heat-induced protein unfolding, interactively visualize results, and collect the relevant parameters. Detailed requirements of the data input format, preprocessing steps, fitting procedure, and visualization are described. One advantage of this approach is that it offers insights into the hydrogen-bonding energies, including both intramolecular and intermolecular contributions within the protein structure and with the surrounding water molecules. To assist users in effectively using and navigating the software tool, example pages and additional resources are also provided. FitFoldData provides researchers in molecular biology and biophysics with a fresh approach to the analysis of protein denaturation studied by CD or DSC and supplies new insights into the roles and energetics of hydrogen bonding in the temperature-dependent structural stability of protein molecules.

## Graphical abstract

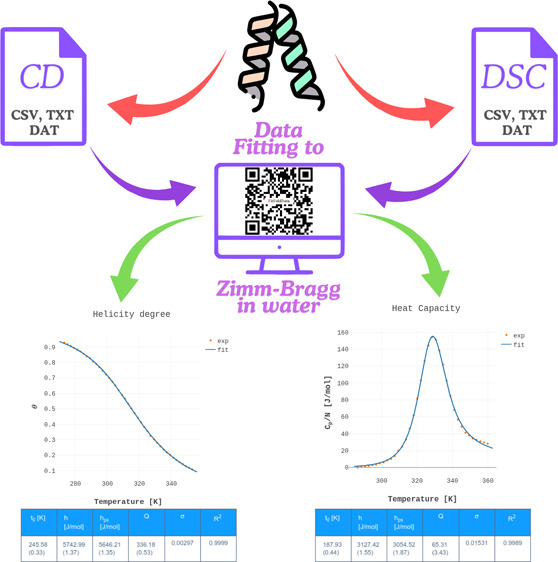

## Introduction

Circular dichroism (CD) and differential scanning calorimetry (DSC) are highly informative experimental techniques that can provide valuable information about the structure, stability, and interactions of biological macromolecules such as proteins, nucleic acids, and lipids.^1,2^ CD is a spectroscopic method that measures the differential absorption of left-handed and right-handed circularly polarized light by chiral molecules.^3^ Since many biological macromolecules exhibit chirality, CD is particularly informative in the study of protein secondary structures, including α-helices and β-sheets.^4–6^ CD can also provide powerful insight into the conformational properties of DNA and RNA structures.^7,8^ DSC is a thermo-analytical technique that directly measures the heat flow in or out of a molecular system in response to temperature-induced structural changes.^9,10^ DSC is widely employed to study the thermal stability of proteins through direct measurements of their melting transition temperature, *T*_m_, which provides unique insights into the overall temperature-dependent stability and energetics of unfolding. Also, DSC measurements reveal temperature-dependent changes in secondary structures of proteins and DNA and can identify specific temperatures at which these structures are disrupted.^11^ Because CD and DSC measurements provide comprehensive information about the structural characteristics and thermal stability of biological macromolecules, they have proven to be indispensable tools in biophysics and molecular biology.

There are relevant preprocessing steps to be applied to both CD and DSC raw data. Namely, in the case of CD, the deconvolution step is crucial: through the comparison with reference spectra for α-helices, β-sheets, and random coils, the entire CD spectrum can be separated into the relative contributions from constituent secondary structures.^12–16^ For DSC, baseline subtraction can seriously affect the final result.^17–19^ However, these preprocessing procedures for both CD and DSC are not related to the thermodynamic models of conformational transitions in proteins and are out of the scope of this report and the tool. Our starting point is the data after the preprocessing step.

In order to clarify the otherwise muddy waters, we also limit ourselves to the single domain proteins, those with one step on the order parameter and one peak on the specific heat graphs.

There is a limited number of thermodynamic models used to process the experimental data on protein folding, the two-state model^20–22^ being the default one, applied to both CD and DSC. The model can provide insights into protein stability as characterized by experimental parameters including the melting temperature (*T*_m_), enthalpy (Δ*H*), and the specific heat (Δ*C*_p_) of unfolding. Zimm–Bragg model^23^ offers an alternative and can also be employed in the analysis of the thermal denaturation monitored by CD and DSC,^24,25^ providing estimates to such key parameters as the *T*_m_ and enthalpy changes (Δ*H*), as well as the cooperativity parameter σ.^26–29^

However, both the two-state and the Zimm–Bragg models lack one very important component, the water. Without water, no protein can function, and not having it in the model used to process experimental data on water solutions of proteins is nonsense. This was the motivation behind the last 15 years of our studies. With the use of a microscopic Hamiltonian representation of the Zimm–Bragg model,^30^ we have shown that effects of solvent (water)^31^ can be incorporated into the model. This approach provides a means to describe and evaluate measurable quantities such as the order parameter and specific heat under the umbrella of a single model.^32–35^ Although possible, such modification comes at a price: formulas for the Zimm–Bragg model in water become somewhat more complicated, as compared to the *in vacuo* model, and the fitting procedure is prone to numerical overflows.

The inherent complexities associated with the application of the aforementioned techniques can pose significant analytical challenges, particularly for data analysis, which often requires specialized equipment and advanced computational methods. In addition, meaningful interpretations of CD and DSC data often require considerable expertise in biophysics, thermodynamics, and statistical analysis. This requirement can render navigation and applications of these techniques difficult for researchers who lack such specialized training. To overcome these challenges, we perceived a critical need for a computational tool that can simplify the analysis and interpretation of CD and DSC thermal denaturation data.

This paper reports on such a tool that combines the results of prior developments of the microscopic formulation of the Zimm–Bragg model with the application of experimental data processing. The unique feature of the reported technique is that it enables the means to extract bonding energy values for intramolecular (N–H···C–O) and intermolecular (N–H···H_2_O or C=O···H_2_O) hydrogen binding, and additional important parameters not accessible with other models. The method was designed to empower researchers in biophysics, molecular biology, and related fields by streamlining the data analysis process and making advanced computational techniques more accessible to a broader audience of users.

In scientific research, enhanced efficiency and precision provided by the advent of advanced computational tools have significantly improved the ability to extract fundamental information from experimental methods.^36–38^ Due to their broad accessibility and user-friendly design, innovative web-based applications have emerged as particularly valuable tools.^39^ In this paper, we present the development of an online tool specifically tailored for the analysis and interpretation of CD and DSC data on protein denaturation. A notable advantage of this tool is that both convenience and accessibility are enabled through open access without the need for registration.

## Methods

The Zimm–Bragg model was first introduced in 1959 to describe the helix–coil transition in polypeptides.^23^ Since its inception, the model has been a mainstay for the analysis of thermal denaturation experiments of biopolymers. The model considers a polypeptide (or DNA) as a linear chain comprised of *N* subunits, *i*, (*i* = 1, *N*), and includes the possibility of nearest-neighbor interactions between adjacent subunits in the chain. In the model, each subunit is assumed to reside in either a coil state or a helix state. Each unit in a coiled state is assigned a statistical weight of 1, while the statistical weight of helix units is assigned a statistical weight, *s*. Nearest-neighbor interactions are included in the model by assigning an additional factor σ for each junction between a coil and a helix unit in the chain. The helix nucleation parameter, σ, accounts for the entropic difference in adding a helix unit next to a coil unit compared with adding a helix unit next to an existing helix unit and extending the helical chain.

In the computational algorithm for the standard Zimm–Bragg model, the characteristic equation and the eigenvalues are given by,

$$\begin{array}{cc} \lambda^{2}-(s+1)\lambda +s(1-\sigma )=0; & \lambda_{1,2}=\frac{1}{2}[1+s\pm \sqrt{(1-s{)}^{2}+4\sigma s}] \end{array}$$ 1

A decade ago, a Hamiltonian formulation of the Zimm–Bragg model was introduced^30^ that enabled inclusion of solvent effects.^31,40^ Recently, this approach was successfully applied to the analysis of thermal denaturation data of proteins measured by CD^32,34^ and DSC.^35^ The seminal feature is the incorporation of specific water interactions into the Zimm–Bragg formalism. This was achieved with the use of a water Hamiltonian that is operative under two conditions: the hydrogen bonds between water molecules and the polypeptide chain can be formed only if internal hydrogen bonds are broken; (ii) water molecules should be in a certain orientation around the N–H or C=O groups of a polypeptide, so that the geometry allows for the hydrogen bond formation. Summing over the degrees of freedom of water in the partition function leads to the renormalization of the stability parameter *s*,^32^ which becomes

$$\tilde{s}(t,t_{0},h,h_{\mathrm{ps}},Q,q)=\frac{1}{Q}\left[{\left(\exp\left[-\frac{h}{R(t-t_{0})}\right]+\frac{\exp\left[\frac{h_{\mathrm{ps}}-h}{R(t-t_{0})}\right]-\exp\left[-\frac{h}{R(t-t_{0})}\right]}{q}\right)}^{-2}-1\right]$$ 2

where *Q* is the entropic cost of hydrogen bonding within the polymer and is a quantity related to the Zimm–Bragg cooperativity parameter σ = 1/*Q*. Yet another entropic parameter, the cost of polymer–solvent hydrogen bonding, is fixed at *q* = 16 for the case of water. *R* is the ideal gas constant, and *t*_0_ is the glass transition temperature that is included due to the non-Arrhenius behavior of the system.^41–45^ *h* and *h*_ps_ are single H-bonding energies within the polypeptide and between the polypeptide and the solvent, respectively. For a more detailed definition of the model and approach, the interested reader is advised to consult refs 31,32,35,40 .

When considering the effects of chain length, the partition function becomes^33,34^

$$Z(\tilde{s},\sigma ,N)=\frac{1-\lambda_{2}}{\lambda_{1}-\lambda_{2}}\lambda_{1}^{N}+\frac{\lambda_{1}-1}{\lambda_{1}-\lambda_{2}}\lambda_{2}^{N}$$ 3

For sufficiently long protein chains, size effects can be ignored, simplifying the partition function to^33^

$$Z(\tilde{s},\sigma ,N)=\lambda_{1}^{N}$$ 4

A central important quantity (order parameter) describing the helix–coil transition in biopolymers is the degree of helicity, defined as the average relative number of H-bonds in individual units comprising the entire chain. It can be calculated as^32,33^

$$\theta (\tilde{s},\sigma ,N)=\frac{\tilde{s}+\sigma }{N}\frac{\partial \ln\;Z}{\partial \tilde{s}}$$ 5

For long chains, inserting eq 4 into eq 5, the degree of helicity is given by,

$$\theta (\sigma ,\tilde{s})=\frac{\tilde{s}+\sigma }{1+\tilde{s}+\sqrt{(1-\tilde{s}{)}^{2}+4\sigma \tilde{s}}}\left(1+\frac{2\sigma -1+\tilde{s}}{\sqrt{(1-\tilde{s}{)}^{2}+4\sigma \tilde{s}}}\right)$$ 6

To evaluate the heat capacity of the system, the internal (average) energy of the system must first be determined according to ref 35

$$E=-\frac{\partial \ln\;Z}{\partial (T^{-1})}$$ 7

The heat capacity is then given by^35^

$$C_{V}=\frac{\partial E}{\partial T}=-2Nh\frac{\partial \theta }{\partial T}-2Nh_{\mathrm{ps}}\frac{\partial X}{\partial T}(1-\theta )+2Nh_{\mathrm{ps}}X\frac{\partial \theta }{\partial T}$$ 8

where

$$X=\frac{\exp[h_{\mathrm{ps}}/T]}{q+\exp[h_{\mathrm{ps}}/T]-1}$$ 9

Substituting eq 9 into eq 8 gives the final expression for the heat capacity:

$$C_{V}=-2Nh\frac{\partial \theta }{\partial T}+\frac{2Nh_{\mathrm{ps}}^{2}\exp[h_{\mathrm{ps}}/T](q-1)}{T^{2}(q+\exp[h_{\mathrm{ps}}/T]-1{)}^{2}}(1-\theta )+\frac{2Nh_{\mathrm{ps}}\exp[h_{\mathrm{ps}}/T]}{\left(q+\exp[h_{\mathrm{ps}}/T]-1\right)}\frac{\partial \theta }{\partial T}$$ 10

For processing DSC data, the approximation that *C*_V_ ≈ *C*_P_ is invoked.

The fitting parameters obtained from the process and their units are displayed in Table 1.

**Table 1:** Fitting Parameters and Their Units

*t*_0_	*h*	*h*_ps_	*Q*
K	J/mol	J/mol	1

## Results and Discussion

### Development and Server of the Web Tool

To ensure security, scalability, and robustness of the online tool, it was built using PHP and the Laravel framework (see Figure 1). Based on the Zimm–Bragg model with solvent effects, the tool facilitates fitting melting data measured by CD or DSC. Fitting results provide important insights into the thermodynamics of conformational stability. The FitFoldData Server is available at https://fit-fold-data.ung.si.

**Figure 1:**
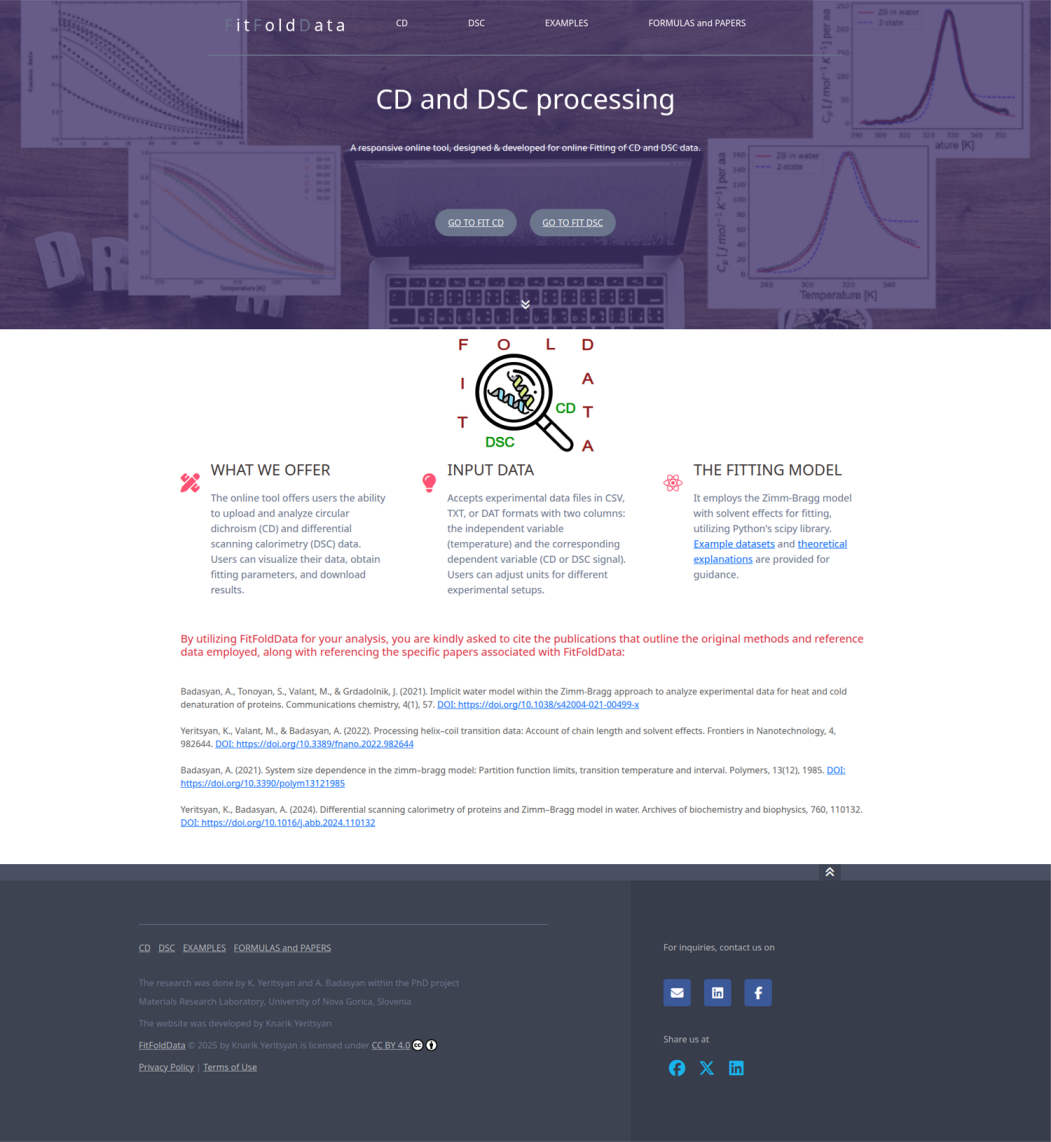
Landing page for the FitFoldData server, available at https://fit-fold-data.ung.si.

### Data Input and Preprocessing

With the forms provided, which include detailed instructions on file preparation and unit adjustment, users can upload experimental data files in various formats, including CSV, TXT, and DAT (Figure 2). Files should not have headers and consist of two columns. The first being the independent variable (e.g., temperature) and the second corresponding to the dependent variable (e.g., CD signal (helicity degree) or heat capacity). For CD data, since the tool fits the order parameter (helicity degree), prior to uploading the CD data files, they should be normalized. For DSC data to remove any background signal, baseline subtraction must be performed before analysis. For analysis of DSC data, the tool will not function properly if the native baseline is not subtracted first. Due to the lack of baseline-subtracted experimental data for cold denaturation in the literature, DSC data analysis of cold denaturation experiments has not yet been implemented.

**Figure 2:**
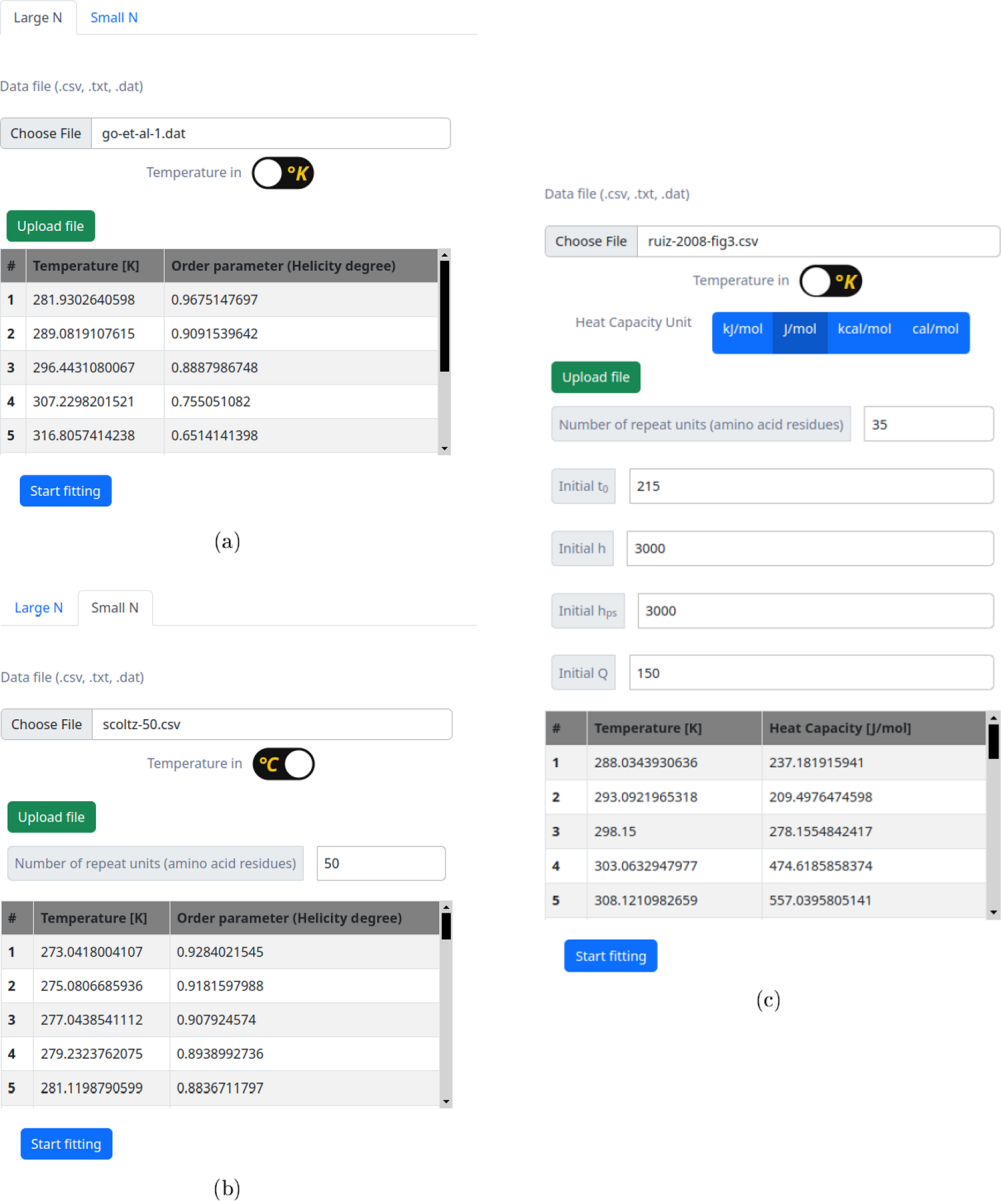
File upload forms for (a) CD long chain, (b) CD short chain, and (c) DSC long chains. These forms provide instructions on uploading files, adjusting units, and setting initial parameter values.

Prior to uploading the online tool, users select the appropriate units for their data. Once uploaded, the tool automatically converts the input data to the appropriate specified units and populates the data table. Before running the fitting procedure, users inspect the table and verify that it is filled correctly, and then click on the appropriate button to proceed.

## Fitting Procedure and Visualization

Fitting is performed in the backend using Python 3,^46^ and the “optimize.curve_fit” function of the “SciPy”^47^ library, which optimizes model parameters that best fit the experimental data. In addition, Python libraries “SymPy”,^48^ “NumPy,”^49^ “Pandas”,^50^ and “JSON”^51^ perform symbolic computations and data manipulation. In some cases, users may be prompted to adjust the initial values of the fitting parameters. For CD data of short chains, the number of units (amino acids) is included in the calculation and must be input. Likewise, for DSC fitting, the number of units must be specified since displayed results are per unit quantities.

The order parameter (degree of helicity) is obtained by fitting the normalized CD signal. For short chains, eq 5 is used and eq 6 is used for long chains.

For computational convenience, instead of the final expression in eq 10, the native baseline-subtracted DSC data are fit using eq 8.

When water is considered as the solvent, the model parameter *q* is set to 16. Fitting parameters include the glass transition temperature (*t*_0_[*K*]), intramolecular (polymer–polymer) H-bonding energy (*h* [J/mol]), intermolecular (polymer–solvent) H-bonding energy (*h*_ps_ [J/mol]), and *Q* is the inverse of the cooperativity parameter σ.

The fitting process begins once a user pushes the ”Start fitting” button. If an error of at least one of the fitting parameters exceeds 50% or the *R* ^2^ values are smaller than 0.5, warning messages alert the user. If the fitting process cannot be successfully completed, then an error message alerts the user to inspect the data and correct the source of the error.

Following completion of the fitting procedure, the tool generates interactive graphs using the “Plotly” JavaScript Open Source Graphing Library. The plotted graphs display both the experimental data points and the fitted theoretical curve. This enables users to visually assess the quality of the fit, as shown in Figure 3. In addition, to facilitate quantitative analysis, a table with values of the fitted parameters along with their percentage errors is displayed. Also, the coefficient of determination (*R* ^2^) is provided, offering a statistical measure of the goodness-of-fit. For future reference, users are given the option to export the results of the fitting procedure, including both the graphs and parameter tables.

**Figure 3:**
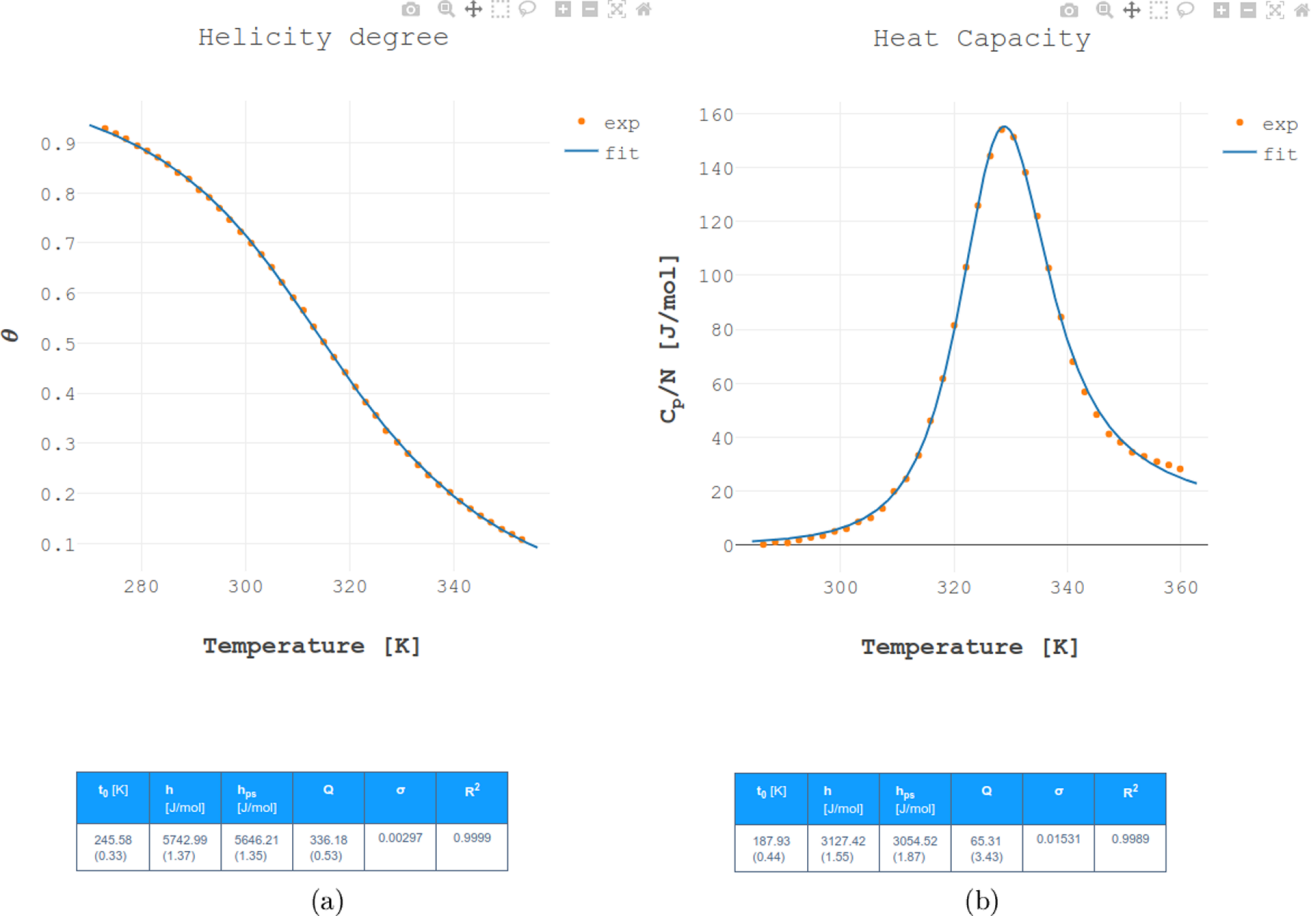
A snapshot of the displayed results. Fitted functions are shown as solid lines, and points correspond to the experimental data. Fitting results are summarized in tables below the graphs; errors of parameters shown in brackets are in percent. Both the graphs and tables can be exported. Depending on the choice of the type of data (CD or DSC), either (a) the temperature dependence of the order parameter (helicity degree), or (b) the temperature dependence of the heat capacity per amino acid will be displayed.

### Example Pages and Resources

To help users understand and utilize the tool, example pages for both CD and DSC results are provided (Figure 4). The preloaded data gathered from the published literature are displayed^24,52–68^ and provide the means to explore the functionality of the tool for real-world examples.

**Figure 4:**
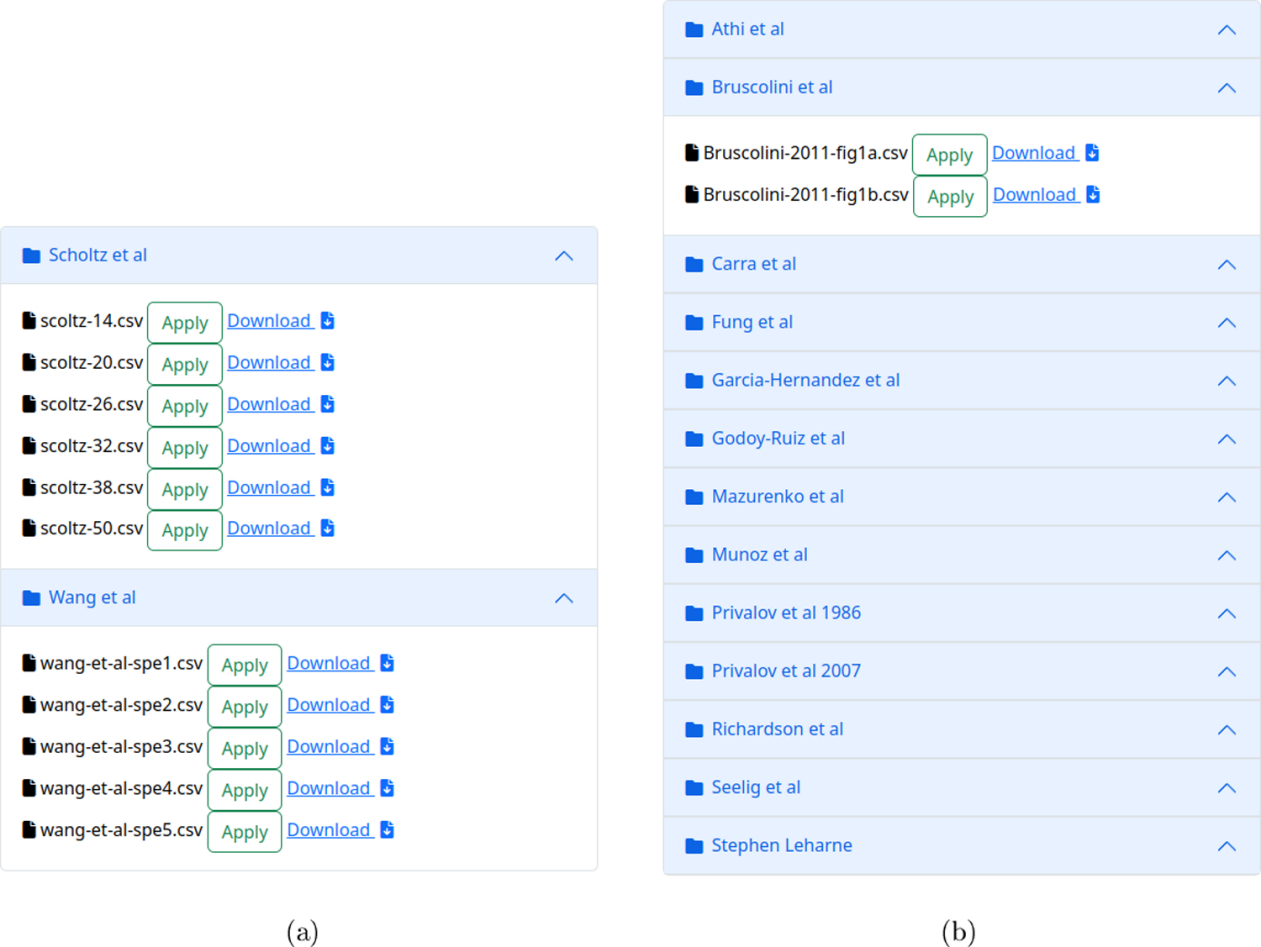
Fitting examples of (a) CD and (b) DSC data. These examples can be utilized, and the corresponding data files are available for download.

For the purpose of data analysis, CD or DSC plots from various publications were digitized in a digital format. This was accomplished using an online digitization tool,^69^ that leverages computer vision technology to accurately extract numerical data from images, such as plots, maps, and other visual materials. The program WebPlotDigitizer combines advanced computer vision algorithms with manual techniques to provide precise data extraction.

In addition, our tool provides a comprehensive set of theoretical sources that offer detailed explanations of the principles and methodologies underlying CD and DSC data analysis. The mathematical notations displayed by the tool are obtained using the “MathJax” JavaScript library, providing for accurate presentation in the web browser.

### Performance Evaluation across Protein Systems

To assess the robustness and generalizability of FitFoldData, we tested the tool on a set of protein unfolding data sets reported in the literature. Although not all data sets explicitly mentioned domain structure, we filtered and selected only those with a single cooperative melting transition (i.e., a single peak), thereby focusing on proteins likely to be single-domain.

The fitting process involves a search in a four-dimensional parameter space. The fitting procedure uses normalized parameters in order to avoid numerical overflow due to the differences of exponents present in formulas (see eq 2). Since fitting is a minimization procedure, due to the search in a rugged multidimensional parameter space, it shows dependence on the initial parameter values. However, by tuning these starting values and monitoring the coefficient of determination (*R* ^2^), we have managed to achieve high-quality fits for all data sets considered. This confirms the convergence stability and reliability of the model implementation. Since the number of fitting parameters is much less than the number of points in each data set, the perfect result cannot be attributed to overfitting.

FitFoldData uses four fitting parameters: *t*_0_ (the glass transition temperature), σ (recalculated as 1/*Q*, related to chain stiffness), and hydrogen bonding energies *h* and *h*_ps_. The *t*_0_ values consistently remained below the lowest experimental temperatures, as expected. The hydrogen bonding energies resulting from these fits fall within the expected range for polypeptides, further supporting the physical relevance of the model outputs. The variability observed in σ (via *Q*) across systems reflects physically meaningful differences in the cooperativity of the protein chains. The consistent convergence across different data sources supports the broad applicability of FitFoldData to various protein classes and experimental setups.

### Validation of Approach

FitFoldData is based on the Zimm–Bragg model in water, a cooperative framework that incorporates solvent interactions into the statistical mechanical modeling of protein unfolding. This model has been previously validated in the publications of other authors,^24^ as well as in our earlier studies,^33–35^ where it was benchmarked against the conventional two-state model. The Zimm–Bragg approach consistently demonstrated superior fitting quality with mean *R* ^2^ values of 0.998 for helicity degree fits (from circular dichroism data) and 0.996 for DSC data.

Furthermore, our earlier work^34^ demonstrated that the model captures finite-size effects and scaling behavior in protein unfolding. As shown in Figure 3 of that study, the fitted experimental curves follow expected trends in transition interval and temperature with respect to reduced chain length, supporting the model’s theoretical assumptions.

In our earlier^35^ and current work, we have also conducted model fits across protein chains measured at different pH values. While not explored in detail here, this opens a promising direction for future research, particularly in analyzing how pH influences the model parameters—such as hydrogen bonding energies and cooperativity factors. Such studies could provide insight into the environmental sensitivity of protein folding thermodynamics and the interplay between solvent conditions and stability.

## Conclusions

Based on the newly formulated Zimm–Bragg model in water, a user-friendly tool named FitFoldData was described. This web-based tool simplifies the analysis of CD and DSC data. It allows efficient data processing and provides valuable insights into the molecular conformation, stability, and thermodynamics of protein molecules. Accessing the tool does not require user registration and enables advanced data analysis for a wide audience.

Future applications could include comparison of fitting results from analysis of both CD and DSC data sets for the same protein under identical conditions. Since the tool uses the same model to analyze both CD and DSC data, researchers can directly compare the unique features reported by each method based on the fitted parameters obtained from both techniques. This direct comparison offers the potential to provide deeper insights into the structural and thermodynamic properties of proteins. Such comparative analyses can divulge formerly unknown correlations between structural characteristics and thermal stability, thereby providing more comprehensive insights into the physical behavior.
